# In Vitro Antifungal Evaluation of Seven Different Disinfectants on Acrylic Resins

**DOI:** 10.1155/2014/519098

**Published:** 2014-06-05

**Authors:** A. Z. Yildirim-Bicer, I. Peker, G. Akca, I. Celik

**Affiliations:** ^1^Department of Prosthodontics, Faculty of Dentistry, Gazi University, Emek, 06510 Ankara, Turkey; ^2^Department of Dentomaxillofacial Radiology, Faculty of Dentistry, Gazi University, 06510 Ankara, Turkey; ^3^Department of Microbiology, Faculty of Dentistry, Gazi University, 06510 Ankara, Turkey

## Abstract

*Objective*. The aim of this study was to evaluate alternative methods for the disinfection of denture-based materials. *Material and Methods*. Two different denture-based materials were included in the study. Before microbial test, the surface roughness of the acrylic resins was evaluated. Then, the specimens were divided into 8 experimental groups (*n* = 10), according to microorganism considered and disinfection methods used. The specimens were contaminated in vitro by standardized suspensions of *Candida albicans* ATCC#90028 and *Candida albicans* oral isolate. The following test agents were tested: sodium hypochlorite (NaOCl 1%), microwave (MW) energy, ultraviolet (UV) light, mouthwash containing propolis (MCP), Corega Tabs, 50% and 100% white vinegar. After the disinfection procedure, the number of remaining microbial cells was evaluated in CFU/mL. Kruskal-Wallis, ANOVA, and Dunn's test were used for multiple comparisons. Mann Whitney *U* test was used to compare the surface roughness. *Results*. Statistically significant difference (*P* < 0.05) was found between autopolymerised and heat-cured acrylic resins. The autopolymerised acrylic resin surfaces were rougher than surfaces of heat-cured acrylic resin. The most effective disinfection method was 100% white vinegar for tested microorganisms and both acrylic resins. *Conclusion*. This study showed that white vinegar 100% was the most effective method for tested microorganisms. This agent is cost-effective and easy to access and thus may be appropriate for household use.

## 1. Introduction

The number of elderly people worldwide has been increased by increasing lifetime [1]. This condition resulted in a high prevalence of edentulism and complete denture wearers [2, 3]. Removable prosthesis may be potential source of several diseases [4]. Oral cavity is colonized by various pathogens and this microbial reservoir can cause several infections including denture stomatitis, aspiration pneumonia, and lung and gastrointestinal infections [5]. Denture stomatitis is possible source of infections in especially immunosuppressed patients [6].

Mechanic and chemical methods are frequently advised for denture hygiene. Some individuals fail to keep oral and denture hygiene because of limited motor capacity, so biofilm accumulation occurred [7]. Arendorf and Walker [8] reported presence of candidiasis in 11% to 67% of complete denture users and* Candida albicans* infections occurred because of poor hygiene. Correct prosthetic use and daily hygiene are important factors for good oral health, greater longevity of the prosthesis, and health of supporting tissue [9]. It was reported that daily hygiene has been essential to prevent oral mucosal inflammation and lesions [10].

It was reported that the patients do not receive professional instructions on how to clean their denture [11]. MacCallum et al. [12] reported that it is difficult for dentists to recommend some sort of cleanser to their patients. Dentures can be cleaned mechanically, chemically, and a combination of them [13]. Denture wearers prefer using the product themselves without information about their benefits or risks [14] and they use homecare products which could cause harmful effects [15]. The most commonly used methods are the use of a brush with hot or cold water and bleach (sodium hypochlorite), diluted 1 : 10 in tap water [13]. Andrucioli et al. [16] reported that the chemical methods were not routinely applied, either due to lack of information or knowledge about these methods, cost or lack of access, or nonavailability of these products in the market. Ideal denture care products should remove inorganic/organic deposits and stains; have cost-effective, bactericidal, and fungicidal properties; and be easy to handle for the human health and harmless for the denture materials [11].

Also, the surface topography of the denture has been shown to greatly influence adhesion and subsequent retention, with more roughened surfaces retaining more organisms [17–19]. Verran and Maryan [17] compared the smooth and rough acrylic surfaces and they reported that* Candida albicans* adhered to rough acrylic surfaces more than smooth surfaces. Also, Quirynen and Bollen [20] reported that rough surfaces like bridges, implant abutments, and denture bases accumulate and retain more dental plaque than smooth surfaces. In clinical practice, internal surfaces of maxillary and mandibular dentures in contact with tissue cannot be polished and these surfaces are rough.

The aim of this study was to evaluate alternative methods for the disinfection of denture-based materials.

## 2. Material and Methods

### 2.1. Preparation of Specimens

In this in vitro study, the tested denture-based materials were a heat-cured poly-(methylmethacrylate) (PMMA) acrylic resin (Acron Duo, Associated Dental Products Ltd., Kemdent, Purton, Swindon, Wiltshire, UK) (*n* = 160) and autopolymerized acrylic resin (Paladent RR, Heraeus Kulzer GmbH, Hanau, Germany) (*n* = 160). To prepare the acrylic samples, a pink modeling wax (Cavex Set Up Modeling Wax, Haarlem, The Netherlands) with dimensions of 10 × 10 × 2 mm was placed in hard dental plaster (Moldabaster S, Heraeus Kulzer GmBH, Hanau, Germany) in a two-part mold using a standard dental flask. The wax was eliminated under running hot water and the plaster surfaces were sealed with one coat of sealant. The heat-cured acrylic denture-based resin was then packed in the molds and the polymerization process was carried out in a water bath at 70°C for 1 h followed by boiling for 30 min for heat-cured acrylic resin. Autopolymerized acrylic resin was packed in the molds and the polymerization process was carried out at 25°C for 10 minutes. All samples were fabricated by one dental technician who makes removable dental prosthesis in Gazi University Faculty of Dentistry for 26 years. In order to give an accurate representation as possible of the tissue surface of the dentures the test sides of the polymerized acrylic resin samples were not polished after remolding. Then, the samples were selected randomly to evaluate the surface roughness of the acrylic resins.

#### 2.1.1. Surface Roughness Evaluation

A surface analyzer (Time TR200, Beijing, China) was calibrated at a sample length of 0.8 mm, 4.0 mm percussion of measure, and 0.5 mm/s and was used to measure the surface roughness (Ra-average roughness) of the resins. Stylus type was diamond with 5 *μ* radius. The stylus was moved across the specimen surface, and three lines were recorded with a distance of 1 mm between each scanning line. The mean Ra was calculated from 3 lines as the mean roughness of the specimen. The resolution of the record data was 0.01 *μ*m.

Then, the samples were only washed with water steam under pressure to remove any possible contaminants present on the surfaces and then stored in distilled water at 37°C for 24 h, prior to adhesion assays and biofilm formation. Acrylic resins were included and 7 different methods were used for acrylic resins disinfection in this study. Firstly, acrylic resins were individually packed for sterilization in autoclave [21].

## 3. Microbiological Process

### 3.1. Preparation of Microorganisms

*Candida albicans (C. albicans) *ATCC#90028 and an oral isolate of* Candida albicans* strains were used in the study. All strains were obtained from the Department of Medical Microbiology culture collection in the School of Medicine at Gazi University. These fungal agents were cultured on Saborraud Dextrose agar (SDA, Merck, Germany) at 37°C for 48 hours aerobically and the inoculums of these fungal agents were adjusted to 2 × 10^8^ CFU/mL (colony forming unit/mL) in the Saborraud Dextrose Broth (SDB, Merck, Germany) according to the 0.5 McFarland test standard turbidometrically and spectrophotometrically also, by using an Elisa reader (Biotek ELx800, USA). After autoclave sterilization, these specimens were divided into 7 experimental groups (*n* = 10), according to* Candida albicans* strains and chemical agents. Standardized suspensions of the chosen microorganisms were adjusted to 2 × 10^8^ CFU/mL and confirmed by measuring their optical density (OD) spectrophotometrically with an Elisa reader (OD: 0.600–0.625). Each acrylic specimen was immersed into test tubes including 10 mL of SDB for* Candida albicans* and an oral isolate of* Candida albicans* individually. Then, they were infected with the 100 *μ*L amount of each of the fungal inoculum as mentioned before and the infected specimens were put into an incubator at 37°C for 24 hours. After the incubation, the specimens were discarded from the tubes gently and washed three times with 5 mL amount of phosphate-buffered saline solution (PBS), (pH: 7.2). The study consisted of 8 groups: 7 experimental and 1 control group (Table 1). Then, experimental groups were individually put into the test tubes including 10 mL chemical products as 1% NaOCl and 50% and 100% white vinegar in sterilized deionized water for 10 min. After time interval, specimens were discarded from the tubes, put into other sterilized test tubes including distilled water, washed for 3 times gently, and put into other test tubes including 10 mL sterilized distilled water. For control group, after infecting the specimens with fungal agents they were put into sterilized test tubes including 10 mL amount of sterilized distilled water. After vortexing for 1 min rigorously all the tubes were diluted as 10^−2^ and 10^−3^ and 25 *μ*L amount of specimen was seeded onto SDA medium for both of the* Candida albicans *strains. After incubation as mentioned before, the grown colonies were counted and the number of the colonies according to the dilution ratio was calculated and defined as CFU/mL.

### 3.2. Data Analysis

The mean values, standard deviations, and medians of the obtained data were calculated with descriptive statistics. The data were statistically analyzed with Kruskal Wallis and Dunn's nonparametrical tests at 95% confidence level for multiple comparisons. Mann Whitney* U* test was used to compare the statistical significance of the roughness of acrylic resins in the study.

## 4. Results

Statistically significant differences (*P* < 0.05) were found between groups of the methods (Groups 1, 2, 3, 4, 5, 6, and 7) and control group (Group 8) for heat-cured acrylic resin according to both* Candida albicans* strains (Table 2). There were statistically significant differences (*P* < 0.05) between groups of the methods (Groups 1a, 2a, 3a, 4a, 5a, 6a, and 7a) and control group (Group 8a) for autopolymerized acrylic resin according to both* Candida albicans* strains (Table 2). The 100% and 50% white vinegar were the most effective for* Candida albicans* ATCC#90028 and following 1% NaOCl, MW, Corega Tabs, UV, and MCP for heat-cured acrylic resin. For* Candida albicans *oral isolate 100% white vinegar was found to be the most effective method following 50% white vinegar, MW, Corega Tabs, UV, MCP, and 1% NaOCl, for heat-cured acrylic resin.

The 100% white vinegar was the most effective for* Candida albicans* (ATCC#90028) following 50% white vinegar, Corega Tabs, MW, 1% NaOCl, UV, and MCP for autopolymerized acrylic resin. For* Candida albicans *oral isolate 100% white vinegar was found to be the most effective method following 1% NaOCl, MW, 50% white vinegar, Corega Tabs, UV, and MCP for autopolymerized acrylic resin. Statistically significant difference (*P* < 0.05) was found between autopolymerised and heat-cured acrylic resins (Table 3). The autopolymerised acrylic resin surfaces were rougher than surfaces of heat-cured resins.

## 5. Discussion

Oral cavity of healthy individuals with or without teeth may be colonized by yeast and bacteria coexisting in a relationship of commensalism [22]. Denture wearing and deficient denture hygiene are the predisposing factors for increasing the number of microorganisms in the oral cavity. So, the bacterial colonization increases and becomes more pathogenic, acting as a potential source of infection [23].* Candida albicans *adhesion to resin materials is promoted by oral environment temperature and the acquired pellicle formed over dentures. Nikawa et al. [24] suggested from their findings appropriate control for denture plaque was essential to the long-term usage of the maxillofacial materials.

Ribeiro et al. [22] found* Candida* spp. (65.5%) more than* Strep. mutans* and* Staph. aureus* on dentures. Also, Baena-Monroy et al. [25] showed the presence of* Candida albicans* on the internal surface of complete dentures.* Candida albicans* is a well-known etiologic agent at denture stomatitis. This inflammatory disorder affects approximately 60% of denture wearers and causes inflammation of the oral mucosa in close contact with the denture [26, 27]. For this reason, we chose* Candida albicans* and oral isolate to determine the better disinfection method for denture-based materials. In addition, the oral mucosa in close contact with the denture (the denture's fitting surface) cannot be mechanically polished and thus presents irregularities and microscopic pores that facilitate bacterial and fungal colonization [28]. Keng and Lim found that plaque levels were significantly higher on the fitting surfaces of the maxillary and mandibular dentures than on the sites of polished surfaces. They reported that this could be due to stagnation, pooling of saliva, and the absence of contact with the tongue on the fitting surfaces [29].

It is admitted that chemical disinfectants are more effective and used easily than mechanical cleaning [30]. Chemical methods have the advantages of being simple to use [31]. Similarly, Palenik and Miller [32] and Salles et al. [33] have found that mechanical cleaning of dentures were insufficient for reducing the number of microorganisms on dentures and palate. Schou et al. [34] reported that 60% of elderly patients, living in shelters, had complete dentures and they generally did not have a habit of cleaning dentures; in addition, financial problems may be important for these individuals in the way of expending any cleanser. Therefore, in the present study, we evaluated the chemical disinfectants on denture material,* Candida albicans.*

Previous studies showed that the analysis of the capacity of denture biofilm removal was based on the internal surface of the complete denture that this surface has greater potential for collection of pathogenic microorganisms [7, 35, 36]. In current study, no finishing and polishing procedures were done in order to simulate the inner surface of a complete denture. Although, statistically significant difference was found between autopolymerised acrylic resin and heat-cured acrylic resin for surface roughness, 100% white vinegar was the most effective method for both of them.

Chemical methods may be recommended for patients with candidiasis to clean the acrylic resin dentures [37]. Nishi et al. [38] reported that daily soaking of dentures in a denture cleanser was effective method for reducing the quantities of microorganisms adhering to dentures. The guidelines outlined by the American College of Prosthodontics recommend that dentures should be cleaned daily by soaking and brushing with on effective, nonabrasive denture cleanser [39]. However, denture weareres who are with limited motor capacity and brushing their dentures may be difficult for them.

NaOCl is widely used as the main root canal irrigant because of its broad antimicrobial activity in endodontic. Although cytotoxic effect of this agent was reported at different concentrations on vital tissues [40], cytotoxic properties of 2–2.5% NaOCl did not appear in short term exposure and no genotoxic effect indicated for host tissues [41] in previous studies. Webb et al. [42] have showed that NaOCl was effective as denture disinfectant and it reduced the adhesion of* Candida albicans* cells on denture acrylic. Also, they have demonstrated that MW was more effective in denture disinfection method than 0.02% and 0.0125% NaOCl. However, Kassab et al. [43] showed that 0.5% NaOCl was more effective than MW energy on acrylic resin and they explained the different results due to the differences in concentration of NaOCl and MW oven watts. NaOCl in concentration 0.5% is effective disinfectant on acrylic resin; however, it is not recommended to disinfect the dentures because of bleaching effect on acrylic denture base [44]. da Silva et al. [45] investigated the effectiveness of disinfectant solutions in the disinfection of acrylic resin specimens contaminated with* Candida albicans, Streptococcus mutans, Staphylococcus aureus, Escherichia coli,* and* Bacillus subtilis*. They found that 1% NaOCl has the best antimicrobial effectiveness against the tested microorganisms [45]. This result is supported by Salvia et al. [46]. Chau et al. [47] reported that a 10 min immersion in 5.25% NaOCl solution is effective for the disinfection of both external and internal surfaces of the acrylic resin. In the present study, although 1% NaOCl provides a significant reduction for* Candida albicans,* MW was more effective than 1% NaOCl for both heat-cured acrylic resin and autopolymerized acrylic resin. This result was in accordance with the results of Kassab et al. [43].

It was reported that MW disinfection is effective and quick which may be a significant advantage for some patients [48]. In addition, previous studies reported that MW irradiation is an effective method to disinfect the acrylic resins. Antimicrobial effect of MW irradiation is showed for removable dentures contaminated with* Candida albicans* for 6–10 min [49]. Some authors [50] suggested denture MW disinfection in water because bubbles released by boiling water help removing microorganisms from the surface, while others have recommended denture disinfection with steam heat in the MW oven [51]. Kassab et al. [43] demonstrated the effectiveness of MW energy in disinfection of acrylic resin denture-based material whether cured by MW or water bath technique. In this study, MW irradiation is used at 650 w for 3 minutes and acrylic resin specimens were put into MW oven without water. It significantly decreased the number of* Candida albicans* and is the most effective method for* Candida albicans* subsequent to 100% white vinegar for autopolymerized acrylic resin and subsequent to 100% and 50% white vinegar for heat-cured acrylic resin. This result is in accordance with previous reports.

Budtz-Jørgensen [52] reported that the advantages of effervescent products are safe and do not damage acrylic resin even when constantly used. Effervescent tablets are classified as chemical soak-type products, and when dissolved in water the sodium perborate readily decomposes to form an alkaline peroxide solution. This peroxide solution subsequently releases oxygen, thereby enabling a mechanical cleaning by the oxygen bubbles in addition to the chemical cleaning [52]. Montagner et al. [14] reported that peroxides products showed different results. The 10 V hydrogen peroxide was efficiently used during 30 min, while Corega Tabs when used for 5 min, as recommended by the manufacturer, did not show the same efficiency. In the current study, the Corega Tabs containing sodium perborate was used for 10 min and it was effective against* Candida albicans* but not as effective as 100% and 50% white vinegar.

UV irradiation has been used for a long time for effective disinfection method of microorganisms [53]. There are several advantages of UV irradiation such as no requirement of any chemicals or heat, and it is fast. It was reported that total counts of bacteria, yeast, and viruses and bacterial counts within saliva were rapidly reduced to very low levels after exposure to UV within the beakers. In addition, the major advantage of UV beakers is that objects are exposed to UV from all directions [54]. Devine et al. [54] indicated that the UV beakers may be useful for disinfection but no sterilization; in many cases, a few colonies of microorganism could be detected after irradiation for 2 min. Also, UV was found to be effective method for fungi on dental impression materials [55] and bacteria on implant materials [56] and dental hand-pieces [57]. Berger et al. [58] used two different UV sanitizers (VIOlight and HIGHDENT) for Gram-negative and Gram-positive bacteria and the devices decreased the amount of bacteria in 83% and 100%, respectively. The results were confirmed by Boylan et al. [59] for VIOlight. Glass and Jensen reported [60] that Pollenex DS60 daily dental sanitizer was effective against bacteria and viruses. Belanger-Giguere et al. [61] reported that application of DenTek UV toothbrush sanitizer for 10 minutes was not as effective against* Streptococcus mutans*. They explained that a longer UV exposure may have eliminated the greater number of microorganisms, but the device used in the study automatically shuts down after 10 minutes. In this study, dental total status vio manuel sanitizer was used for 20 minutes by adjusting manually according to the manufacturer's recommendation and the device significantly decreased the number of* Candida*, both heat-cured acrylic resin and autopolymerized acrylic resin.

Although the white vinegar is not frequently used in dentistry as a disinfectant [45], it is preferred as a promising alternative disinfectant in several areas because of its low toxicity and low cost [62]. White vinegar was frequently used in 50% and 100% concentrations to disinfect toothbrushes and acrylic resins [45]. da Silva et al. [45] reported that the white vinegar showed effective antimicrobial activity against* Candida albicans* and* Staphylococcus aureus* in 100% concentration for acrylic resins as 1% NaOCl and 2% glutaraldehyde. This result was supported by Salvia et al. [46] and they remarked that this agent was as effective as 1% NaOCl and 2% chlorhexidine digluconate against* Candida albicans*,* Escherichia coli,* and* Streptococcus mutans. *On the contrary, Komiyama et al. [63] investigated the effectiveness of 50% white vinegar for toothbrush disinfection and it was found to be effective for* Staphylococcus aureus*,* Streptococcus mutans,* and* Streptococcus pyogenes,* but not for* Candida albicans*. In this study, white vinegar was used in 50% and 100% concentrations for 10 min. Both of them were found to be considerably effective for* Candida albicans *both heat-cured acrylic resin and autopolymerized acrylic resin. Interestingly, white vinegar 100% was the most effective method for* Candida albicans*.

Propolis is a complex mixture of several resinous substances [64] known as a safe natural bee product and has been used in folk medicine since early times especially in Europe due to its antimicrobial, antioxidant, and anti-inflammatory properties [65]. It has been shown that propolis can be used to treat* Candida *infections [66]. It has been stated that propolis has received the attention of clinicians and researchers due to its diverse pharmacological activities and low toxicity [67]. de Castro et al. [64] evaluated the antifungal activity of propolis on* Candida albicans*, and they reported that propolis demonstrated fungicidal action against all three* Candida albicans *morphogenetic types. Previous studies showed that propolis is an effective product and alternative chemical mouthwash against various oral microorganisms due to being nontoxic and natural product [68]. Also, propolis containing mouthrinses ready for use is available in the markets. In this study, a ready to use mouthwash is applied because of easy accessible. There is no study related with these products in the literature; the mouthrinses containing propolis which are laboratory-manufactured were used in previous studies. Although statistically significant difference was found between MCP and control group, MCP was the least effective agent against* Candida albicans* in this study.

## 6. Conclusion

In the present study, different denture disinfection methods were used for* Candida albicans *strains. White vinegar 100% was found to be the most effective agent against both* Candida albicans* strains. This agent is cost-effective and easy to access and it may be appropriate for household use. However, white vinegar is relatively new in dentistry and may be unknown by many clinicians. Further studies determining all of the effects, including the biocompatibility or toxic effects of white vinegar, may increase clinicians' awareness about its antimicrobial capacity, and it might also be introduced to other fields of dentistry, such as root-canal treatment. Laboratory-manufactured MCP was used in previous studies. There is no study about ready-to-use MCP for denture disinfection. In this study, MCP was found to be the least effective agent for* Candida albicans.* Therefore, further studies are necessary to determine the efficacy of different trademarked ready-to-use MCP.

## Figures and Tables

**Table 1:** The methods used in this study.

Groups	Methods	Time (minutes)
Group 1	Heat-cured acrylic resins were immersed in 1% NaOCl	10
Group 2	Heat-cured acrylic resins were placed in MW oven on high power (650 watt)	3
Group 3	Heat-cured acrylic resins were placed in UV sanitizer on high power	20
Group 4	Heat-cured acrylic resins were immersed in MCP	10
Group 5	Heat-cured acrylic resins were immersed in white vinegar 50%	10
Group 6	Heat-cured acrylic resins were immersed in white vinegar 100%	10
Group 7	Heat-cured acrylic resins were immersed in Corega Tabs (potassium monopersulfate, sodium bicarbonate, sodium lauryl sulfoacetate, sodium perborate monohydrate, and sodium polyphosphate)	10
Group 8 (control)	Heat-cured acrylic resins were washed with tap water	1
Group 1a	Autopolymerized acrylic resins were immersed in 1% NaOCl	10
Group 2a	Autopolymerized acrylic resins were placed in MW oven on high power (650 watt)	3
Group 3a	Autopolymerized acrylic resins were placed in UV sanitizer on high power	20
Group 4a	Autopolymerized acrylic resins were immersed in MCP	10
Group 5a	Autopolymerized acrylic resins were immersed in white vinegar 50%	10
Group 6a	Autopolymerized acrylic resins were immersed in white vinegar 100%	10
Group 7a	Autopolymerized acrylic resins were immersed in Corega Tabs	10
Group 8a (control)	Autopolymerized acrylic resins were washed with tap water	1

**Table 2:** Statistical assessment of antifungal effects of disinfectants on both acrylic resin materials.

Type of prosthesis	Candida strains	Methods	Mean	Standard deviation	Minimum	Maximum	Kruskal Wallis	*P* value	Statistically significant difference
Heat-cured acrylic resin	*C. albicans* ATCC#90028	1% NaOCl	840.00	1.490.12	0	4.600	54.494	0.000*	*Control—1% NaOCl *Control—50% white vinegar *Control—100% white vinegar *Control—Corega Tabs *Control—MW *Control—UV *Control—MCP
		50% white vinegar	0.00	0.00	0	0			
		100% white vinegar	0.00	0.00	0	0			
		Corega Tabs	1.696.00	4.160.52	0	13.400			
		MW	1.442.00	3.208.05	0	10.400			
		UV	2.720.00	5.321.82	0	16.800			
		MCP	9.318.00	9.122.75	1.680	23.800			
		Control	17.630.000.00	23.872.766.91	6.500.000	82.000.000			
	*C. albicans* oral isolate	1% NaOCl	69.840.00	164.890.10	0	535.200	58.368	0.000*	*Control—1% NaOCl *Control—50% white vinegar *Control—100% white vinegar *Control—Corega Tabs *Control—MW *Control—UV *Control—MCP
		50% white vinegar	60.00	134.99	0	400			
		100% white vinegar	0.00	0.00	0	0			
		Corega Tabs	3.974.00	8.327.66	0	21.400			
		MW	624.00	1.688.78	0	5.400			
		UV	5.880.00	10.105.75	0	32.000			
		MCP	31.750.00	24.627.77	2.400	84.000			
		Control	3.668.000.00	1.355.358.09	2.200.000	6.240.000			

Autopolymerized acrylic resin	*C. albicans* ATCC#90028	1% NaOCl	404.00	643.24	0	1.800	56.732	0.000*	*Control—1% NaOCl *Control—50% white vinegar *Control—100% white vinegar *Control—Corega Tabs *Control—MW *Control—UV *Control—MCP
		50% white vinegar	120.00	269.98	0	800			
		100% white vinegar	0.00	0.00	0	0			
		Corega Tabs	274.00	359.51	0	800			
		MW	264.00	572.00	0	1.600			
		UV	1.769.00	3.778.12	0	12.200			
		MCP	15.500.00	13.630.77	3.600	42.000			
		Control	2.761.000.00	605.207.95	1.890.000	3.780.000			
	*C. albicans* oral isolate	1% NaOCl	85.00	197.27	0	600	61.720	0.000*	*Control—1% NaOCl *Control—50% white vinegar *Control—100% white vinegar *Control—Corega Tabs *Control—MW *Control—UV *Control—MCP
		50% white vinegar	1.067.00	2.747.61	0	8.800			
		100 % white vinegar	0.00	0.00	0	0			
		Corega Tabs	8.180.00	16.323.52	0	42.000			
		MW	279.00	363.33	0	800			
		UV	12.316.00	17.104.91	180	54.000			
		MCP	2.284.500.00	3.235.882.54	145.000	8.400.000			
		Control	3.854.000.00	1.698.078.65	1.780.000	6.700.000			

*Statistically significant difference *P* < 0.05.

**Table 3:** Surface roughness (Ra) values of heat-cured and autopolymerized acrylic resins.

Materials	*N*	Mean	Standard deviation	Median	Minimum	Maximum	Mann Whitney *U*	*P* value
Heat-cured acrylic resin	10	1.27	0.20	1.20	1.01	1.57	17.000	0.013*
Autopolymerised acrylic resin	10	1.76	0.48	1.71	1.02	2.38		

*Statistically significant difference was found between heat-cured and autopolymerised acrylic resin (*P* < 0.05).
